# CAN NEIGHBORHOOD AND LOCAL ENVIRONMENTS MODIFY COGNITIVE DECLINE? FINDINGS FROM THE REGARDS STUDY

**DOI:** 10.1093/geroni/igz038.100

**Published:** 2019-11-08

**Authors:** Philippa J Clarke, Jessica M Finlay

**Affiliations:** 1 University of Michigan, Ann Arbor, Michigan, United States; 2 Social Environment and Health, Institute for Social Research, University of Michigan, Ann Arbor, Michigan, United States

## Abstract

Environmental factors may significantly increase the risk of, or buffer against, age-related cognitive decline, yet policies and practices to improve cognitive health outcomes to date largely overlook the role of neighborhoods and socio-physical environmental contexts. Residence in socioeconomically advantaged neighborhoods may promote cognitive function through greater density of physical and social resources (e.g., libraries, parks, coffee shops, air conditioning, community centers) that promote physical activity, facilitate mental stimulation, and encourage social engagement. This symposium will identify natural, built, and social environmental factors linked to changes in cognitive function over time (assessed by animal naming and world list learning tests) based on secondary data analyses of a national, racially diverse (42% Black), population-based sample of over 30,000 Americans aged 45+ in the Reasons for Geographic and Racial Differences in Stroke (REGARDS) study followed annually since 2003. The first two papers investigate the roles of racial residential segregation and education on cognitive function disparities at the neighborhood and city scale. The third paper explores fast-food restaurants as socially interactive community spaces for older adults that may help buffer against cognitive decline. The fourth paper investigates effects of local air temperature on cognitive testing performance, and discusses how regional differences and seasonality may buffer or exacerbate temperature-cognition associations. Altogether, the symposium elucidates how cognitive health is impacted by a complex interplay of individual and geographic factors. The papers inform policy-making efforts to improve physical neighborhood environments and social community contexts, which are critical to the well-being of older adults aging in place.

